# Mechanisms of post-transcriptional gene regulation in bacterial biofilms

**DOI:** 10.3389/fcimb.2014.00038

**Published:** 2014-03-25

**Authors:** Luary C. Martínez, Viveka Vadyvaloo

**Affiliations:** Paul G. Allen School for Global Animal Health, Washington State UniversityPullman, WA, USA

**Keywords:** biofilm, post-transcriptional regulation, RNA-binding proteins, ncRNAs, riboswitch, toxin-antitoxin systems, RNases, c-di-GMP

## Abstract

Biofilms are characterized by a dense multicellular community of microorganisms that can be formed by the attachment of bacteria to an inert surface and to each other. The development of biofilm involves the initial attachment of planktonic bacteria to a surface, followed by replication, cell-to-cell adhesion to form microcolonies, maturation, and detachment. Mature biofilms are embedded in a self-produced extracellular polymeric matrix composed primarily of bacterial-derived exopolysaccharides, specialized proteins, adhesins, and occasionally DNA. Because the synthesis and assembly of biofilm matrix components is an exceptionally complex process, the transition between its different phases requires the coordinate expression and simultaneous regulation of many genes by complex genetic networks involving all levels of gene regulation. The finely controlled intracellular level of the chemical second messenger molecule, cyclic-di-GMP is central to the post-transcriptional mechanisms governing the switch between the motile planktonic lifestyle and the sessile biofilm forming state in many bacteria. Several other post-transcriptional regulatory mechanisms are known to dictate biofilm development and assembly and these include RNA-binding proteins, small non-coding RNAs, toxin-antitoxin systems, riboswitches, and RNases. Post-transcriptional regulation is therefore a powerful molecular mechanism employed by bacteria to rapidly adjust to the changing environment and to fine tune gene expression to the developmental needs of the cell. In this review, we discuss post-transcriptional mechanisms that influence the biofilm developmental cycle in a variety of pathogenic bacteria.

## Introduction

During their life cycles bacterial pathogens must often transit between different habitats and have to respond to continually changing environmental conditions. Rapid adaptation to these changing conditions is a key factor for survival and replication. Some bacterial pathogens exhibit multicellular behaviors as a conserved strategy for long-term bacterial survival in nature and during infections. One of these multicellular behaviors is biofilm formation (Mah and O'Toole, 2001; Matz and Kjelleberg, 2005; Anderson and O'Toole, 2008). Biofilm represents a mode of growth that enables bacteria to establish persistent relationships with their surroundings providing protection against environmental stressors, antibiotics, predation, and host immunity (Stoodley et al., 2002). This phenomenon has been observed in diverse Gram-negative and Gram-positive bacterial species. Although mixed-species biofilms predominate in most environments, single-species biofilms exist in a variety of infections. To understand the role of biofilm formation in infections there has been notable research focused on pathogenic biofilm-producer organisms in such diverse genera as *Pseudomonas*, *Vibrio*, *Escherichia*, *Salmonella*, *Listeria*, *Streptococcus*, *Staphylococcus*, *Yersinia*, and *Mycobacteria*.

Biofilm formation has a significant impact in medical and industrial settings. The formation of biofilm on many medical and technological devices may cause severe complicating problems affecting human health and industrial processes. The growth of bacterial biofilm on human tissues results in chronic infections which are challenging for antimicrobial therapies because they are extremely resistant to antibiotic treatment. This is primarily due to the increased prevalence of dormant cells, known as persisters, within the biofilm (Lewis, 2005; Hatt and Rather, 2008; Hall-Stoodley and Stoodley, 2009). This negative impact of biofilm has stimulated research aimed to identify specific components of the physical biofilm structure and regulatory aspects of the process of biofilm development toward creating anti-biofilm strategies (Sommer et al., 2013). On the other hand, despite the detrimental impact of biofilm, they are useful in engineering applications and in many natural settings where they are favored for promoting beneficial microbial associations (Currie, 2001; Singh et al., 2006; Kreth et al., 2008). Understanding the mechanisms of biofilm formation can therefore lead to its manipulation for either its enhancement or eradication. With the recent advances in molecular biology, understanding the underlying molecular basis of biofilm formation has become possible and this provides novel opportunities to disrupt/enhance biofilm formation.

## Biofilm structure: what does it take to form a biofilm?

**Figure 1:**
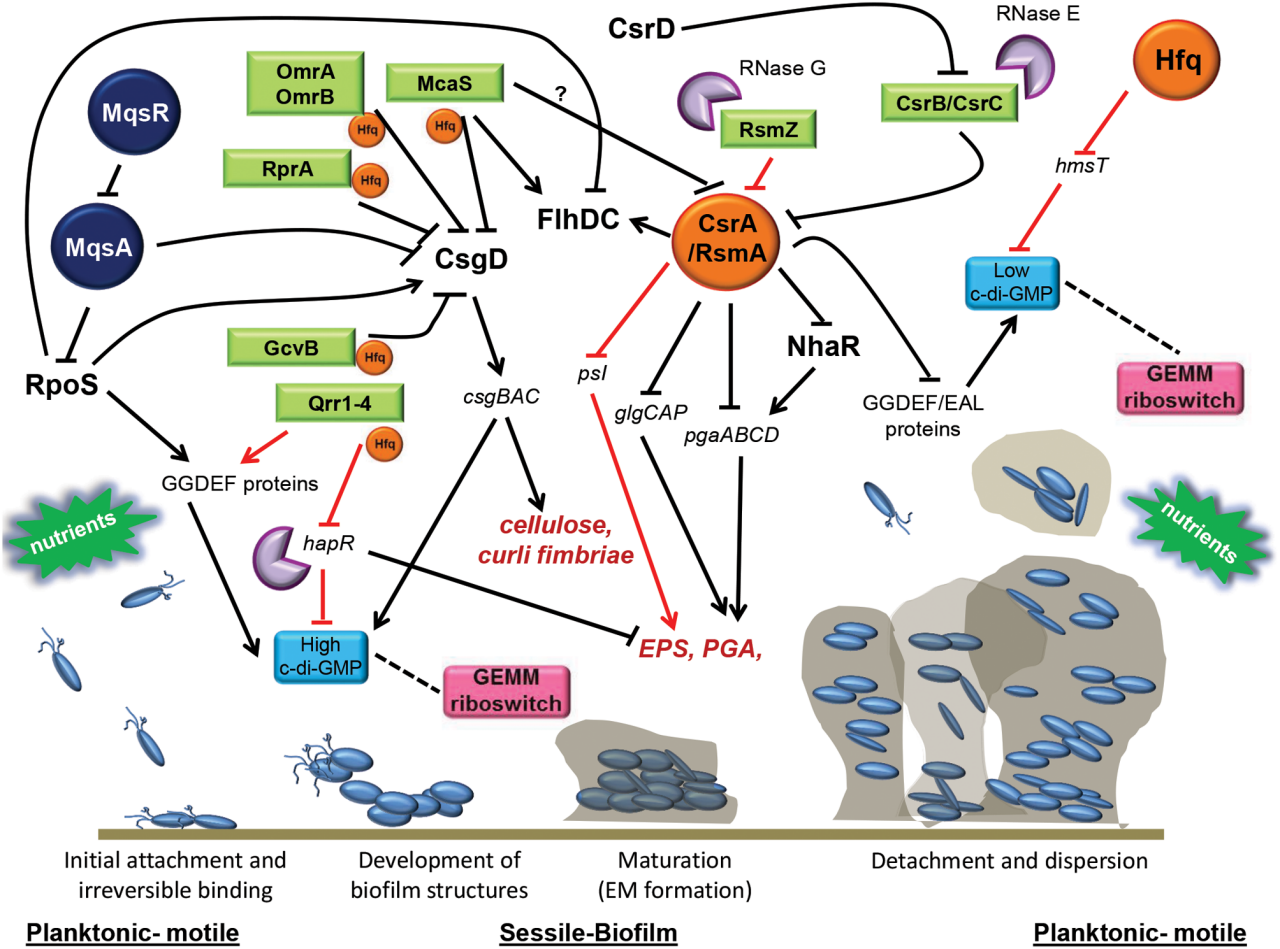
**Post-transcriptional regulatory networks directing biofilm formation**. Numerous post-transcriptional regulatory factors, mainly affecting biofilm maturation at the level of synthesis or exopolysaccharides has been described currently and are represented here. The **RNA-binding protein**, CsrA, represses biofilm formation by directly and indirectly affecting stability of mRNA transcripts encoding important polysaccharides constituting the extracellular biofilm matrix. CsrA acts alternately by repressing GGDEF/EAL encoding proteins which determine **c-di-GMP levels**, or by favoring motility, rather than biofilm formation, by stabilizing transcripts encoding the master regulator of flagella, FlhDC. Several **ncRNAs** (OmrA/B, RprA, GcvB, McaS) regulate biofilm by repressing *csgD*, encoding the CsgD master regulator involved in the production of curli fimbria, cellulose, and c-di-GMP. Other ncRNAs (CsrB/CsrC, McaS) favor biofilm formation by blocking CsrA activity (e.g., in *E. coli* and *Salmonella*) or by positively affecting production of c-di-GMP (Qrr1-4) and expression of exopolysaccharides (e.g., *V. cholerae*). To exert their function, many of these sRNAs need to be bound to the RNA-binding chaperone Hfq, which has been reported to also repress biofilm by affecting the expression of the GGDEF protein, HmsT (*Y. pestis*), required to synthesize c-di-GMP. Changes in the levels of c-di-GMP are sensed by the GEMM **riboswitch** which leads to regulation of organelle biosynthesis that promotes the transformation between motile and sessile lifestyles, where increased c-di-GMP leads to increased biofilm formation and *vice versa*. The **RNases** E and G mainly cause the decay of ncRNAs that are involved in the biofilm formation. Degradation of the antitoxin *mqsA* transcript by the MqsR toxin, leads to inhibition of motility, and induction of *csgD*, favoring both curli and cellulose production. Environmental factors such as nutrient concentrations (glucose, amino acids) and other physiological stresses (osmolarity, pH, oxidative stress, antimicrobials) are important signals mediating the switch from the planktonic motile to sessile biofilm lifestyles. RNA-binding proteins are shown in orange, ncRNAs are shown in green, riboswitches are shown in pink, TA systems are shown in dark blue and RNases are shown in violet. CsgD, FlhDC, NhaR, and RpoS are major transcriptional regulators. Cellulose, PGA and EPS are expolysaccharides. Hard arrows indicate a direct or indirect positive effect, while truncated arrows indicate a direct or indirect negative effect. Black arrows show those mechanisms that are present in *E. coli* and are shared in many other pathogens, while red arrows show those mechanism that are present in specific microorganisms (see text for details).

The ability to form biofilm is a universal attribute of several bacteria but the mechanism that different bacterial species employ to produce biofilm may vary according to the specific strain attributes and the diverse environments they occupy. Even though some biofilm structural components can be recognized as common features, their chemical compositions may vary. The process of biofilm formation is dynamic and complex but the stages of development seem to be conserved among a remarkable range of prokaryotes and typically involve the attachment to a surface by planktonic bacteria, replication, cell-cell adhesion to form microcolonies, maturation, and detachment (represented in Figure 1). Because these steps overlap at some point, the growth cycle of a biofilm is described in three general stages here:

### Initial attachment and development of biofilm structures

This first stage initiates with reversible attachment of bacteria to a favorable surface and is highly dependent on the physicochemical and electrostatic interactions between the bacterial envelope itself and the substrate. Attachment occurs seconds after the bacterial cells detect required environmental signals including changes in nutrients and nutrient concentrations (glucose, indole, polyamines), inorganic molecules (iron, phosphate), pH, antimicrobials, temperature, oxygen concentration, osmolarity, and host derived signals (bile acids, hydrogen peroxide) (O'Toole and Kolter, 1998; Aparna and Yadav, 2008; Karatan and Watnick, 2009). At this point bacterial cells usually exhibit a logarithmic growth rate.

Attachment is facilitated by different adhesive organelles, e.g., flagella and type IV pili play important roles in surface aggregation in *Pseudomonas* spp. and *Vibrio cholerae*, whereas fimbriae like type 1 pili, curli, and conjugative pili are important for biofilm formation in *Escherichia coli* (Thelin and Taylor, 1996; O'Toole and Kolter, 1998; Watnick and Kolter, 1999; Jackson et al., 2002). Curli fimbriae are also produced by other enteric bacteria such as *Shigella*, *Salmonella*, *Citrobacter*, and *Enterobacter* (Smyth et al., 1996). Following initial attachment, bacterial cells multiply to form aggregated microcolonies and can inter-communicate by producing quorum sensing molecules, which is one of the key events leading to biofilm development in some bacteria (Camilli and Bassler, 2006).

### Maturation

During the maturation stage cell aggregates begin to grow in layers in a three-dimensional matrix (Aparna and Yadav, 2008). The maturation stage still requires adhesive organelles, however, this stage is mostly characterized by cell-to-cell interactions and formation of important surface components that contribute to the structure of the biofilm (McLean et al., 1997; Davies et al., 1998; Holden et al., 1999; Pesci et al., 1999; Whiteley et al., 1999; De Kievit et al., 2001).

A general hallmark feature that determines the mature biofilm architecture is the presence of the extracellular matrix (EM) surrounding the resident biofilm bacteria. Besides its crucial role in maintaining biofilm structure, it enables bacteria to remain in close proximity to each other, protects embedded bacteria from desiccation, acts as a diffusion barrier, and allows bacteria to evade recognition by the host immune system. The biofilm matrix generally consists of up to 97% water, 2–5% microbial cells, 3–6% extra-polymeric substances (EPS) and ions (Sutherland, 2001). The EPS may account for 50–90% of the total organic carbon of biofilm and this is primarily composed of exopolysaccharides, but it also includes proteins (extracellular proteins and enzymes), DNA and RNA, which constitute less than 2% of the biofilm matrix (Flemming and Wingender, 2001; Sutherland, 2001; Donlan, 2002; Flemming et al., 2007). The polysaccharide composition along with other components such as proteins usually varies among different bacteria and even between strains of a single species, although there are some common polysaccharides produced by multiple species of bacteria. It has been proposed that after contact of bacteria with a surface, altered gene expression induces changes that initiate synthesis of extracellular polysaccharides since alginate, the EPS of *P. aeruginosa* biofilms, is up-regulated in recently attached cells in comparison with planktonic cells (Davies and Geesey, 1995). The systematic three dimensional development of mature *V. cholerae* biofilms following attachment, and specifically as this is related to synthesis of the EPS, *Vibrio* polysaccharide (VPS) and the three major EM proteins, RbmA, RbmC, and Bap1, has been captured in real time in elegant work done using advanced microscopy (Berk et al., 2012).

One of the most common and extensively studied matrix exopolysaccharides is the poly–N-acetylglucosamine (PGA or PNAG) that is utilized to construct the biofilm matrices (Wang et al., 2004; Izano et al., 2007, 2008; Parise et al., 2007) and is produced by diverse bacterial species, including *E. coli*, *Staphylococcus epidermidis*, *Staphylococcus aureus*, *Yersinia pestis*, *Actinobacillus* spp., *Aggregatibacter actinomycetemcomitans*, and *Bordetella* spp. Some bacteria are capable of producing multiple polysaccharides which usually confer different physiological properties to the biofilm matrix, e.g., *P. aeruginosa* makes biofilms constructed from 3 distinct exopolysaccharides (alginate, Pel, and Psl), *Bacillus subtilis* secretes 2 polymers (EPS and PGA) while *E. coli* principally synthesizes PGA, colanic acid, and cellulose. Cellulose is a commonly produced polymer among the enteric pathogens including *Salmonella*, *Citrobacter*, *Enterobacter*, and *Shigella*, where it has been strongly associated with the ability to form a rigid biofilm (Sutherland, 2001; Zogaj et al., 2001, 2003; Solano et al., 2002; Spiers et al., 2003; Da Re and Ghigo, 2006; Ude et al., 2006; Lopez et al., 2010).

The protein fraction of the EPS is generally quite large and an important component for biofilm formation that include structural proteins like adhesins, and other cell surface associated proteins like pili, flagella, curli and amyloid fibers (Prigent-Combaret et al., 2000; Toledo-Arana et al., 2001; Gohl et al., 2006; Flemming et al., 2007; Larsen et al., 2007), as well as a homologous group of large proteins, referred to as biofilm-associated proteins, found in, e.g., *Staphylococcus*, *Enterococcus*, *Vibrio*, and *Salmonella* spp. (Cucarella et al., 2001; Kristich et al., 2004; Latasa et al., 2006; Fong and Yildiz, 2007; Berk et al., 2012). The production of 3 biofilm-associated matrix proteins (RbmA, RbmC, and Bap1) and the *Vibrio* polysaccharide (VPS) are involved in *V. cholerae* biofilm formation, where the RbmA protein is specifically involved in cell-to-cell adhesion and Bap1 facilitates adherence of the biofilm to surfaces (Berk et al., 2012).

Extracellular DNA (eDNA) may act as a structural component of the biofilm matrix, where it can be found in varying quantities. eDNA is a major structural component in the biofilm matrix of *S. aureus* (Izano et al., 2008). Its role in biofilms was confirmed in *P. aeruginosa*, where DNAse was added to the culture medium and this resulted in dissolution of preformed biofilms (Whitchurch et al., 2002; Nemoto et al., 2003; Bockelmann et al., 2006, 2007). In fact, it has been reported that eDNA facilitates the self-organization of bacterial biofilm, as it coordinates the movement of cells and is required for their assemble into the intricate network of furrows that form the biofilm (Gloag et al., 2013).

### Detachment and dispersion

In this stage these sessile communities (surface-attached) can give rise to planktonic (free-floating) bacteria that can rapidly multiply and disperse to colonize new surfaces. Some studies have shed light on the signals and signaling networks that lead to dispersal of biofilms. Most often the nutritional status of the environment dictates bacterial behavior, including the biofilm dispersal response that can result from both decreases and increases in environmental nutrients (Anderl et al., 2003; Walters et al., 2003; Sauer et al., 2004; Gjermansen et al., 2005; Thormann et al., 2005, 2006). Besides nutrient availability, there are other factors influencing the biofilm dispersion, such as the presence of oxygen, by-products of anaerobic metabolism, quorum sensing signaling, and levels of the chemical second messenger molecule cyclic-di-GMP (c-di-GMP). The exact mechanistic details of dispersal have not been elucidated. However, among the factors that influence biofilm detachment are synthesis of enzymes that degrade extracellular polymeric substances in the biofilm matrix, release of EPS and surface-binding proteins, induction of motility, surfactant production and cell lysis, hydraulic shear, sloughing, and erosion (Boyd and Chakrabarty, 1994; Lee et al., 1996; Allison et al., 1998; Jackson et al., 2002; Stoodley et al., 2002; Kaplan et al., 2003, 2004; Webb et al., 2003; Boles et al., 2005; Itoh et al., 2005; Purevdorj-Gage et al., 2005; Chambless and Stewart, 2007).

The processes described above for biofilm development are not necessarily synchronized throughout the whole biofilm, but are often localized so that at any time a small area on the surface may contain biofilm at each developmental stage. Even when cells in a biofilm are clonal populations, it has been shown that there are subpopulations of phenotypically dissimilar cell types, and that this cell heterogeneity is a consequence of alterations in gene expression in response to extracellular conditions encountered by the cells within a microenvironment (Lopez et al., 2010). Therefore, since the synthesis and assembly of biofilm matrix components is a costly and an exceptionally complex process, the transition from the planktonic state to the sessile state requires the coordinate expression and simultaneous regulation of many genes by complex genetic networks involving all levels of gene regulation.

## Mechanisms of post-transcriptional regulation

The regulation of gene expression can potentially occur at several stages during the transfer of information from a gene to its protein product. As for many other bacterial processes, transcriptional regulation is perhaps the most well studied form of controlling biofilm production. However, post-transcriptional regulation provides a powerful way for the bacteria to rapidly adjust to the changing environment and to fine tune gene expression to the needs of the cell. Post-transcriptional regulation can be formally defined as the control of gene expression that occurs after transcription but ahead of translation. In this review we will focus only on those mechanisms that influence biofilm formation by acting at a post-transcriptional level, some of which are summarized in Figure 1.

The post-transcriptional regulatory mechanisms controlling biofilm formation include mainly the activity of RNA-binding proteins, *cis*- and *trans*-acting small non-coding RNAs, and RNases. Due to the multifactorial nature of biofilm formation, some of these mechanisms act directly or indirectly to both spatially and temporally coordinate production of the components integral to biofilm development. This is achieved by regulating the intracellular levels of c-di-GMP and by controlling genes affecting the production of major adhesive and aggregative factors, e.g., pili, flagella and fimbriae, cell surface and intercellular matrix components or production of exopolysaccharides that constitute the biofilm EM.

### C-di-GMP: a central molecule to switch from planktonic to sessile mode of life

Central to the mechanism of post-transcriptional regulation of biofilm formation is a tiny cyclic RNA chemical second messenger molecule, c-di-GMP. C-di-GMP is implicated in controlling various cellular functions including virulence, motility, and adhesion, although its principal role is controlling the switch from motile planktonic lifestyle to the sessile biofilm forming state. Elevated intracellular levels of c-di-GMP promote synthesis of exopolysaccharides and enhanced auto-aggregation and surface adhesion leading to biofilm formation (Simm et al., 2004). In contrast, reduced intracellular c-di-GMP concentrations are associated with decreased biofilm formation. The mechanistic details of how post-transcriptional gene regulation is mediated by the c-di-GMP molecule will be discussed below in section Riboswitches.

C-di-GMP is synthesized by GGDEF motif containing proteins that encode diguanylate cyclase (DGC) enzyme activity required to convert two molecules of GTP to c-di-GMP. Degradation of c-di-GMP is carried out by EAL or HD-GYP motif containing-proteins that encode phosphodiesterase activity leading to hydrolysis of c-di-GMP to pGpG (Ross et al., 1987; Tal et al., 1998; Romling et al., 2005). The GGDEF or EAL/HD-GYP protein encoding genes controlling c-di-GMP synthesis are present in multiple copies in bacterial genomes especially in those pathogens that infect multiple hosts, e.g., *E. coli* encodes 34, *Salmonella enterica* serovar Typhimurium encodes 27, *V. cholerae* encodes 63 (Galperin et al., 2001; Galperin, 2004) and *P. aeruginosa* encodes 39 such genes with GGDEF, EAL, or HD-GYP domains in their genomes (Kulasakara et al., 2006). Thus, it stands to reason that the c-di-GMP signal transduction is tightly synchronized and regulated to avoid interference between the functionally distinct c-di-GMP responsive systems.

Such distinct environmental niche-dependent regulation by c-di-GMP is exemplified in control of biofilm formation central to transmission of *Y. pestis* biofilm from the foregut of its flea vector. *Y. pestis*, the agent that causes bubonic plague, contains 10 genes encoding DGCs and PDEs in its genome (Sun et al., 2011). However, only two of the DGC enzymes, encoded by *hmsT* and *y3730*, have been shown to be functional in controlling synthesis of c-di-GMP to activate production of biofilm while only one phosphodiesterase (PDE), encoded by *hmsP*, is active and degrades c-di-GMP, reducing biofilm synthesis (Kirillina et al., 2004; Bobrov et al., 2005; Sun et al., 2011). Interestingly, despite their similar expression patterns during varied *in vitro* growth conditions and their equally robust expression in the flea gut, the production of biofilm *in vitro* appears to be mainly dependent on expression of *hmsT* while biofilm formation within the flea gut is mainly dependent on *y3730* expression. The Y3730 DGC alone appears to promote autoaggregation indicating a potential role for this DGC in additional biofilm promoting functions (Sun et al., 2011). Additionally, Y3730 responds to a specific flea environmental cue detected by the gene product of the co-transcribed gene *hmsC* (Ren et al., 2013). The different predominant roles for each of these DGCs are driven by post-transcriptional control mechanisms still yet to be fully defined.

Another well-characterized example of how distinct phenotypes can be regulated by specific DGCs is found in *P. aeruginosa*. Mutational studies of the numerous DGCs encoded in the genomes of *P. aeruginosa* strains, PAO10 and PA14, indicate that while some DGCs impact biofilm formation, others either appear not to affect biofilm formation, or may require specific *in vivo* activating signals to initiate their regulation of biofilm formation; and yet other DGCs are essential for functions such as cytotoxicity and virulence (Kulasakara et al., 2006). Nevertheless, there is a correlation between increased amounts of c-di-GMP and biofilm formation in *P. aeruginosa*. Interestingly, unique localized pools of c-di-GMP may be found in the cell due to differential subcellular localization of DGCs and this may be intimately connected with specific signaling to cognate gene targets to produce biofilm (Merritt et al., 2010; Massie et al., 2012). This observation was made in *P. aeruginosa* when mutations in 2 DGCs, SadC and RoeA, showed insignificant changes in total intracellular c-di-GMP quantities despite their negative effects on biofilm formation. RoeA and SadC appeared to be differentially localized in the cell thus producing unique localized pools of c-di-GMP that likely specifically activate different gene targets for EPS production or increased swarming motility, to negatively impact biofilm formation (Merritt et al., 2010).

### Regulatory RNA binding proteins

This group contains those proteins known to bind to mRNA, and by doing so regulate translation initiation, mRNA stability, and the half-life of the message. Below we will outline the functions of the most thoroughly studied RNA binding proteins CsrA and Hfq which are able to post-transcriptionally regulate biofilm formation in various bacterial pathogens.

#### CsrA

CsrA (RsmA) proteins are a family of RNA binding proteins that are widely distributed central components of the global carbon storage regulatory system (Csr) involved in the control of many cellular functions and virulence traits, like motility, quorum sensing, carbon metabolism, interaction with hosts and biofilm production (Altier et al., 2000; Lenz et al., 2005; Heroven et al., 2008; Brencic and Lory, 2009). Homologs of CsrA proteins have been found throughout the prokaryotic world, in both Gram-negative and Gram-positive bacteria, although the majority of CsrA functional studies have been described in Gram-negative bacteria (White et al., 1996). Several bacterial genomes encode more than one CsrA homolog, such as *Legionella pneumophila*, *Pirellula*, *Pseudomonas fluorescens*, and *Coxiella burnetti* (Mercante et al., 2006). CsrA proteins control the expression of target genes at a post-transcriptional level by various methods: binding sequences overlapping the Shine–Dalgarno (SD) sequence in target mRNAs, occluding ribosome binding and translation, and enhancing mRNA degradation (Liu et al., 1995; Liu and Romeo, 1997; Baker et al., 2002, 2007; Lucchetti-Miganeh et al., 2008; Timmermans and Van Melderen, 2010). The CsrA proteins have been shown to repress biofilm formation post-transcriptionally in several different ways described below (and exemplified in Figure 1).

***Direct effects on extracellular polysaccharide production***. In *E. coli*, the major extracellular polysaccharide that is produced in biofilm formation is poly-β-1,6-N-acetyl-D-glucosamine (PGA) (Wang et al., 2004; Itoh et al., 2005). The *pgaABCD* gene locus is required for the synthesis of this biofilm polysaccharide adhesion PGA that promotes attachment, cell-to cell adherence and stabilization of biofilm structure (Wang et al., 2004). CsrA represses *pga* gene expression and the production of PGA by binding specifically to the transcript of the *pgaA* gene. This prevents ribosome binding, affecting *pgaABCD* mRNA stability and promoting accelerated degradation of this transcript (Wang et al., 2005). Consequently, in a *csrA* mutant, the biofilm production is increased and in agreement with this, an insertional inactivation of *csrA* has no effect on biofilm in the absence of *pgaC* (Wang et al., 2004, 2005). Interestingly, it has been demonstrated that the effect of a *csrA* mutant on biofilm formation can successfully be restored by CsrA of *Campylobacter jejuni*, even though they exhibit variability in the amino acids important for RNA binding and are therefore substantially divergent (Fields and Thompson, 2012). This demonstrates both similarities and differences in both Csr systems and somewhat different mechanisms of action of CsrA among the ε- and γ-proteobacteria, as the former lacks several genes in the CsrA pathway (Kulkarni et al., 2006; Fields and Thompson, 2012).

In *P. aeruginosa*, Psl is a major extracellular polysaccharide and therefore one of the major structural components of the biofilm extracellular matrix. RsmA, the CsrA homolog, binds to *psl* mRNA and upon this binding initiates *psl* mRNA folding into a secondary stem-loop structure that blocks the SD sequence, preventing subsequent ribosome access and protein translation (Irie et al., 2010) and biofilm formation.

***Indirect effects on extracellular polysaccharide production***. CsrA can repress *E. coli* biofilm synthesis by affecting *pgaABCD* transcription via translation inhibition of the LysR-type transcriptional regulator NhaR. NhaR responds to the Na concentration and pH which are factors that activate *pgaABCD* (Goller et al., 2006). CsrA then binds to the mRNA of *nhaR* and outcompetes ribosomal binding at the translation initiation region, thus blocking *nhaR* mRNA translation and consequently repressing the transcription of *pgaABCD* and in turn biofilm production (Pannuri et al., 2012).

Perhaps the primary and best-known effect of CsrA on biofilm formation is through its regulatory control of glycogen metabolism. CsrA interacts with the leader mRNA of the *glgCAP* operon, that includes the glycogen biosynthetic genes *glgC* and *glgA* and the gene encoding the catabolic enzyme *glgP* (Yang et al., 1996; Baker et al., 2002). By inhibiting *glgCAP* expression, CsrA represses glycogen synthesis and turnover, both processes necessary for optimum biofilm formation in the *Enterobacteriaceae*, since in *Salmonella* and *E. coli*, glycogen levels are observed to be positively correlated with biofilm formation (Yang et al., 1996; Bonafonte et al., 2000; Jackson et al., 2002). Glycogen is used for the generation of precursors of PGA (Wang et al., 2004) and the role of CsrA in controlling glycogen metabolism indirectly affects optimal biofilm production.

***Indirect effects by inducing motility***. There is a negative correlation between the expression of the flagella and the induction of biofilm formation. While flagella play a role in initiating initial contact with a favorable surface, they also promote motility. In *E. coli*, CsrA can bind and stabilize the transcript of *flhDC*, which encodes the master regulator of flagella synthesis (Wei et al., 2001). Thus, CsrA also indirectly represses biofilm by promoting motility through positive regulation of the flagella master regulator, FlhDC (Wei et al., 2001). In agreement with this, expression of flagella decreases upon attachment, which correlates with the decreased expression of *csrA* once the bacteria start to grow on surfaces. Flagella expression is later reactivated once the biofilm matures, leading to resumption of motility (Pratt and Kolter, 1998), which correlates with CsrA overexpression and leads to biofilm dispersal (Jackson et al., 2002).

***Effect on GGDEF/EAL proteins***. Until recently, CsrA was thought to affect biofilm formation only through repression of glycogen metabolism and via its positive regulatory effect on the swimming-motility master regulator *flhDC* (Wei et al., 2001). However, it was recently found that CsrA can also affect biofilm production by controlling the expression of proteins with GGDEF/EAL motifs by binding to them causing their destabilization and degradation. This is the first example of post-transcriptional control of c-di-GMP metabolizing proteins mediated through their mRNA stability (Jonas et al., 2008). Consistent with this, mutants of *csrA* have increased c-di-GMP levels.

CsrA negatively controls biofilm in a c-di-GMP-dependent way, as it down-regulates the expression of several genes encoding GGDEF/EAL proteins. Although some of these proteins do not have orthologs in bacteria that also contain CsrA homologs, the link between CsrA and c-di-GMP levels could be a conserved trait, since it is present in *E. coli, Salmonella* and *Pseudomonas* (Jonas et al., 2008, 2010). In *E. coli*, CsrA represses the expression of at least 29 genes encoding GGDEF/EAL domain proteins, two of them, the GGDEF proteins YcdT and YdeH, encode DGCs that also inhibit motility, hence CsrA enhances motility and inhibits biofilm (Jonas et al., 2008). In the case of *Salmonella*, CsrA controls the expression of 8 genes encoding GGDEF/EAL motifs by both direct and indirect mechanisms (Jonas et al., 2010).

*Xanthomonas campestris* is the causative agent of black rot disease of cruciferous plants (Ryan et al., 2011). In these bacteria, RsmA (CsrA) represses biofilm production by binding to the transcripts of 3 genes encoding GGDEF domain proteins. A mutation in *rsmA* is associated with elevated intracellular levels of c-di-GMP and increased biofilm. Mutation in *rsmA* is also associated with decreased expression of *manA*, which encodes the biofilm dispersing enzyme mannanase, and increased expression of *xag*, a gene encoding a glycosyl transferase, required for biofilm formation. However, these effects on *xag* and *manA* are indirect and work through the c-di-GMP-responsive regulator Clp (Lu et al., 2012).

***Activation of biofilm dispersal***. In preformed mature *E. coli* biofilms, the induction of CsrA expression causes release of viable planktonic cells from the biofilm, reflecting a role for CsrA in activation of biofilm dispersal (Jackson et al., 2002). Interestingly, this effect can be overridden in the presence of glucose and confirms the importance of nutrients as signals in both biofilm formation and its dispersal (Jackson et al., 2002).

#### Hfq

Hfq is a chaperone RNA-binding protein required for the virulence of many pathogenic bacteria that plays a pivotal role in the post-transcriptional regulation of large numbers of genes (Chao and Vogel, 2010). Once Hfq binds to an mRNA, it can either stabilize or promote its degradation (Vytvytska et al., 1998; Masse et al., 2003; Meibom et al., 2009). Hfq has a key role in the regulatory function of non-coding small RNA (ncRNAS) molecules as it can bind to them thereby stabilizing interactions between the ncRNA and its target mRNA as well as protecting them from RNase degradation (Moller et al., 2002; Vecerek et al., 2003; Zhang et al., 2003; Vogel and Luisi, 2011). Hfq is involved in regulation of biofilm produced by many bacteria, where it mostly positively regulates biofilm production.

In *Yersinia pestis*, the causative agent of plague, Hfq is required for growth of some *Y. pestis* strains and efficient biofilm formation when bacteria are grown in TMH, a defined medium that promotes biofilm production (Rempe et al., 2012). In these bacteria biofilm formation is necessary to block the foregut of fleas, the transmission vector for plague (Jarrett et al., 2004; Hinnebusch, 2005). Only those fleas that are blocked due to the presence of a *Y. pestis* biofilm in the foregut can transmit the disease to other hosts. It was observed that a mutant in *hfq* is affected in biofilm-mediated blockage formation during flea gut infection (Rempe et al., 2012) indicating that Hfq, is an important factor mediating transmission of the bubonic plague bacteria from fleas.

In contrast, when *Y. pestis* is grown in rich brain heart infusion medium (BHI), Hfq represses biofilm formation. This was demonstrated by showing that absence of *hfq* leads to an increase in biofilm formation in these conditions (Bellows et al., 2012). The negative effect of Hfq on biofilm formation is due to its inverse control on the abundance of HmsT and HmsP, the DGC and PDE enzymes responsible for the synthesis and degradation of c-di-GMP, respectively (Bellows et al., 2012). Hfq directly negatively regulates HmsT at a post-transcriptional level, by binding to the *hmsT* transcript and decreasing its stability and half-life, thus decreasing c-di-GMP levels and biofilm formation (Bellows et al., 2012). It also contributes to the positive regulation of HmsP at a transcriptional level perhaps by an indirect mechanism as the absence of Hfq does not affect the stability of *hmsP* mRNA (Bellows et al., 2012).

Uropathogenic *E. coli* (UPEC) are the primary cause of urinary tract infections (UTIs), and it has been proposed that these bacteria form a biofilm to adapt to the poor nutrient environment present in the urinary tract (Stanley and Lazazzera, 2004). Their persistence in the urinary tract therefore depends not only on their ability to invade host epithelial cells and multiply intracellularly, but also on their ability to form a biofilm (Bower et al., 2005; Soto et al., 2006). Hfq influences a number of virulence-related UPEC phenotypes, including biofilm formation and is critical to the ability of UPEC to effectively establish and persist within the urinary tract. Mutants in *hfq* are negatively affected in their ability to produce biofilm and consequently a persistent bacterial UTI (Kulesus et al., 2008). The different regulatory RNAs that could be interacting with Hfq to produce these effects remain to be elucidated.

Similarly to UPEC, Hfq acts to increase biofilm formation in other pathogens such as *Salmonella enterica* Typhimurium, *V. cholerae*, *P. fluorescens*, *Stenotrophomonas maltophilia*, *Erwinia amylovora*, and *Vibrio alginolyticus*. The effects of *hfq* mutation in these pathogens are not only restricted to the biofilm production; they often exhibit pleiotropic phenotypes, including defects in quorum sensing, motility, antibiotic susceptibility, growth rate, stress tolerance, and virulence (Hammer and Bassler, 2007; Kint et al., 2010; Wu et al., 2010; Liu et al., 2011; Monteiro et al., 2012; Roscetto et al., 2012; Zeng et al., 2013).

The effect of Hfq on biofilm formation seems to be dependent on the different growth conditions, which could reflect the distinct arsenal of ncRNAs that may be interacting with Hfq under different environmental conditions. Most of the Hfq-associated ncRNAs have not been identified but recent studies in *E. coli* reported that the ncRNAs McaS, RprA, GcvB, OmrA/B, ArcZ, and SdsR, are implicated in the regulation of CsgD, the major biofilm regulator (Boehm and Vogel, 2012; Jorgensen et al., 2012; Mika et al., 2012; Thomason et al., 2012).

### Non-coding RNAs

The ncRNAs are a large group of small molecules of RNA (50–500 nts in length) that are not translated into proteins. They act as important trans-acting regulators of gene expression in bacteria, directly influencing protein synthesis at the post-transcriptional level (Gottesman and Storz, 2011) especially under stress conditions. These molecules carry out their regulatory function by base-pairing to a limited complementary sequence on the mRNAs of their cognate target gene, which leads to changes in mRNA translation or stability or both, thereby influencing the target gene expression. The ncRNAs can act by activating or repressing gene expression depending on the portion of the mRNA molecule they base-pair with (Waters and Storz, 2009). Employing ncRNAs to fine-tune the expression of genes, stabilize mRNA, and regulate biofilm formation is an energetically economical strategy (Waters and Storz, 2009). Synthesis of ncRNA is part of the on-going transcription process and likely does not require any additional enzymatic processing as far as is known, therefore defeating the requirement for additional proteins to be synthesized. This should be especially advantageous under limiting conditions of stress that normally trigger biofilm production.

CsrB and CsrC, are two well-studied examples of ncRNAs in *E. coli* that contain many CsrA-binding sites and function by antagonizing CsrA activity by binding and sequestering this protein, counteracting its translational repression activity (Liu et al., 1997; Romeo, 1998; Weilbacher et al., 2003; Babitzke and Romeo, 2007; Heroven et al., 2008; Lucchetti-Miganeh et al., 2008; Timmermans and Van Melderen, 2010). The study of CsrB and CsrC as regulatory factors for gene expression in bacteria has greatly increased in the recent years. Orthologs of CsrB/CsrC (and CsrA), have been identified in many other gammaproteobacteria (Lapouge et al., 2008), including *Salmonella enterica* (CsrB/CsrC, and CsrA) (Martinez et al., 2011), *Pseudomonas* species (RsmX/RsmY/RsmZ, and RsmA) (Kay et al., 2005), *Vibrio* species (CsrB/CsrC/CsrD, and CsrA) (Lenz et al., 2005), *Erwinia carotovora* (RsmB, and RsmA) (Cui et al., 2008), and *Yersinia* species (CsrB/CsrC and CsrA) (Heroven et al., 2012), where they also control the expression of many genes required for a wide variety of cellular functions, including biofilm (Lucchetti-Miganeh et al., 2008; Timmermans and Van Melderen, 2010). The role of these ncRNAs in biofilm production is at a post-transcriptional level by controlling the levels of free CsrA/RsmA protein resulting in activation of biofilm formation (Figure 1). It has been demonstrated that CsrB and CsrC have a redundant function, which means that CsrB levels exhibit a compensatory increase in response to CsrC disruption and *vice versa* (Weilbacher et al., 2003). In agreement with this, it has been shown in *E. coli* and *Salmonella* that only mutants lacking both *csrB* and *csrC*, but not the single mutants in *csrB* or *csrC*, show reduction in biofilm production (Wang et al., 2005; Teplitski et al., 2006). However, other unidentified ncRNAs may act similarly to CsrB/CsrC to counteract the effects of CsrA. This has been demonstrated by the ability of CsrA from *C. jejuni* to restore the affected CsrA phenotypes in *E. coli*, even though homologs to CsrB and CsrC or other proteins involved in the Csr pathway in *E. coli* have not been identified in *C. jejuni* (Parkhill et al., 2000; Hofreuter et al., 2006; Kulkarni et al., 2006; Fields and Thompson, 2012). It has been proposed that these two ncRNAs are in turn post-transcriptionally controlled by CsrD, a GGDEF/EAL protein that binds CsrB and CsrC leading to their decay by converting them to substrates for RNAse E degradation (Suzuki et al., 2006).

Although ncRNAs are widespread in nature and an increasing number of ncRNAs have been found to regulate critical pathways, the specific functions of many ncRNAs are still unknown. However, there is growing evidence that could support their role in biofilm production. These molecules often require the chaperone protein Hfq for their expression, stability, and/or function. The effect of Hfq on biofilm production varies among different bacteria and their conditions of growth, suggesting that Hfq-dependent ncRNAs specific to different environments could be the determining factors required to produce biofilm. Accordingly, studies have attempted to identify and characterize the arsenal of ncRNAs in different pathogens and under different conditions by using deep sequencing approaches and have found distinct predominating ncRNAs under different conditions. The ncRNAs that are produced in *Y. pestis*, for example, are distinct depending on growth environment alterations, e.g., temperature, medium and *in vivo* vs. *in vitro* (Koo et al., 2011; Beauregard et al., 2013; Yan et al., 2013).

It has been reported that iron concentrations can regulate biofilm formation via ncRNAs in the periodontal pathogen *A. actinomycetemcomitans*. Some ncRNAs whose expression is dependent on the ferric uptake regulator Fur, which functions to repress genes involved in iron uptake in iron-rich environments, were identified in this pathogen. It is proposed that these ncRNAs could be interacting with biofilm-associated genes, including the *flp* fimbrial operon and genes associated with EPS, according to *in silico* models (Amarasinghe et al., 2012). So far the ability of these iron-regulated ncRNAs to modulate biofilm formation has been demonstrated, but their functional target genes remain to be elucidated (Amarasinghe et al., 2012).

The ncRNAs are sometimes part of complex regulatory networks that finally induce biofilm formation by post-transcriptional control. In *V. cholerae* there are 4 redundant ncRNAs, called Qrr1-4 that are under control of the quorum sensing response (Lenz et al., 2004). They repress translation by binding and occluding the RBS of several mRNAs, including *hapR*, which encodes HapR, a transcription factor that controls the expression of genes involved in biofilm formation (Bardill et al., 2011). Each Qrr is predicted to base pair to the 5'-UTR of *hapR* aided by Hfq (Lenz et al., 2004). HapR indirectly represses the expression of the exopolysaccharide biosynthesis operon and alters the intracellular levels of c-di-GMP (Hammer and Bassler, 2003, 2009). Furthermore, Qrr ncRNAs may also facilitate degradation of *hapR* mRNA by cellular RNases as increased levels of the ncRNAs correlated with decreased levels of *hapR* transcript (Svenningsen et al., 2008). On the other hand, the ncRNAs Qrr1 and Qrr2 have been shown to base-pair to the 5'-UTR of the *vca0939* gene to positively control its translation to a GGDEF motif containing protein and synthesis of c-di-GMP (Hammer and Bassler, 2007). It had been assumed that the four Qrr ncRNAs act in a similar way to induce the synthesis of Vca0939 as the Qrr/*vca0939* pairing occurs through a region that is 100% conserved among them, which interestingly is the same region required to base-pair to the 5'-UTR of *hapR* (Zhao et al., 2013). All these studies demonstrate how biofilm formation is linked to the detection of cell density by multiple pathways, where at high density biofilm formation would be repressed because none of the Qrr ncRNAs would be present to repress HapR (Bardill et al., 2011).

We are beginning to understand that post-transcriptional control involving ncRNAs is highly complex and there is growing evidence for a role of these molecules as crucial regulators, alongside well-known global transcriptional regulators. Perhaps the current best characterized example of just how extremely complex and convoluted a post-transcriptional regulatory network can be is that of the regulatory network of CsgD, the global transcription factor that integrates signals to control biofilm formation in *E. coli* and *Salmonella*. CsgD induces the expression of the *csgBAC* operon, required for the production of curli fimbriae, and cellulose, two major adhesive factors required for biofilm, as well as the production of c-di-GMP. Furthermore, CsgD represses flagellar operons favoring the biofilm-formation phenotype (Ogasawara et al., 2011). CsgD can be post-transcriptionally repressed by 5 discrete Hfq–dependent small RNAs (OmrA, OmrB, GcvB, McaS, RprA) that respond to various stress conditions (Boehm and Vogel, 2012). These small RNAs directly target the 5'-UTR of the *csgD* mRNA hence blocking translational initiation. The ncRNAs OmrA and OmrB, are induced by the two component regulator, OmpR-EnvZ during high osmolarity conditions (Holmqvist et al., 2010). The multicellular adhesive ncRNA McaS, present only in *E. coli*, *Enterobacter* and closely related species, is an important small RNA element that disfavors biofilm by binding and exerting inverse control on *csgD* and *flhCD* mRNAs (Thomason et al., 2012). In the absence of McaS, *csgD* upregulation occurs; alternately, increased McaS expression activates the synthesis of *flhDC*, encoding the flagellar master regulator (Thomason et al., 2012; Jorgensen et al., 2013). In contrast to its occlusion of the SD region in the 5'-UTR of the *csgD* mRNA, McaS is able to unfold the stem-loop structure of the *flhDC* mRNA thus exposing the hidden SD sequence for translation initiation (Thomason et al., 2012). However, the McaS-dependent regulation of biofilm seems to be complex, as it also activates PGA, leading to the formation of biofilm that is independent of the CsgD-pathway. McaS-mediated activation of *pga* requires the presence of CsrA as McaS can be immunoprecipitated with CsrA in an *E. coli* lysate and the McaS transcript has several GGA motifs which are recognized targets for binding to CsrA (Holmqvist et al., 2010; Holmqvist and Vogel, 2013; Jorgensen et al., 2013). McaS is therefore the first example of a ncRNA that regulates gene expression through both CsrA and Hfq RNA binding proteins, acting by two different mechanisms: base-pairing and protein titration (Jorgensen et al., 2013).

RprA is the small RNA that responds to envelope stress mediated through the phosphorelay system Rcs proteins. RprA appears to fine tune expression of *csgD* by targeting several branches of the CsgD network in response to environmental cues (Mika et al., 2012). RpoS, the general stress response sigma factor is activated by RprA and in turn activates *csgD* expression and curli biosynthesis once cells transition into stationary phase, while RprA inhibits *csgD* expression through the DGC YdaM. RprA appears to modulate synthesis of colonic acid in the presence of curli and also cellulose biosynthesis, possibly to balance the expression of these EPS matrix components as necessitated by a specific environmental cue (Mika et al., 2012). RprA is another example of a ncRNA that plays a dual role to repress biofilm formation. The fifth known small RNA, GcvB is expressed in response to amino acid availability to repress *csgD* under these specific nutritional conditions (Boehm and Vogel, 2012; Jorgensen et al., 2012).

In *Salmonella typhimurium*, LuxS, the synthase enzyme required in quorum sensing response, is involved in biofilm production, as it has been shown that a *luxS* deletion mutant is impaired in biofilm production. This defect can be restored upon complementation only with *luxS* along with its native promoter region. This lead to the finding that adjacent to the *luxS* coding sequence is a ncRNA called MicA whose balanced concentration is essential for proper biofilm formation (Kint et al., 2010). The mechanism by which MicA exerts its function is still unknown (Kint et al., 2010).

### Riboswitches

Riboswitches are structured non-coding RNA domains that form part of the mRNA usually at the 5'-UTR where they can act in *cis* to control gene expression upon selectively binding ligands (Mandal and Breaker, 2004; Coppins et al., 2007; Roth and Breaker, 2009). Two main domains constitute the riboswitch: an aptamer molecule, which senses and binds a single ligand and an expression platform, usually located downstream of the aptamer that switches its secondary structure according to its ligand binding status to direct gene expression, by either transcriptional or translational mechanisms (Mandal and Breaker, 2004; Weinberg et al., 2007; Roth and Breaker, 2009). The mechanisms of riboswitches sensing and binding a metabolite are categorized into families and classes that are dependent on the required ligand, together with the secondary structure that is formed upon ligand binding.

The mechanism of post-transcriptional gene regulation by the c-di-GMP molecule is facilitated by recognition of a riboswitch. Here c-di-GMP acts as a ligand and mediates its regulation by binding to a riboswitch class in mRNA called GEMM (genes for the environment, for membranes, and for motility). This particular conserved RNA domain GEMM, is located upstream of genes encoding DGC and PDE proteins, as well as in other genes that are controlled by c-di-GMP (Sudarsan et al., 2008; Smith et al., 2010). The GEMM motif was first described in *V. cholerae* which carries two sequences of GEMM. One of the well-studied c-di-GMP GEMM riboswitches, Vc1, appears to regulate *gbpA* which encodes a chitin-binding protein required for adherence to chitin and epithelial cells and required for mammalian infection (Kirn et al., 2005; Sudarsan et al., 2008). The second Vc2 is located upstream of the *tfoX*-like gene *vc1722* (Sudarsan et al., 2008), that has been shown to be up-regulated in *V. cholerae* mutants with the rugose phenotype, characterized in part by increased biofilm production (Lim et al., 2006; Beyhan et al., 2007).

### Toxin-antitoxin systems

Bacterial biofilms contain an increased prevalence of dormant cells known as persisters, which are characterized by up-regulation of genes known as toxin-antitoxin (TA) modules. These TA systems typically consist of two genes organized in an operon that encodes a stable toxin that disrupts an essential cellular process and a labile antitoxin (either RNA or protein) that prevents toxicity by binding to the toxin and forming a tight complex that neutralizes the toxin (Van Melderen and Saavedra De Bast, 2009). Depending on the nature of the interacting molecules, TA systems can be divided in 3 groups. In the Type I group, the antitoxin is a transcript that is antisense to the toxin mRNA and pairing of the two RNAs promotes mutual degradation. In the Type II group, both toxin and antitoxin are proteins and together they form a complex that masks the activity of the toxin. In the Type III group the toxin is a protein which is inhibited by an antitoxin RNA (Blower et al., 2011). Although the mechanism of toxicity at the molecular level is slightly different, the Type II toxins, MqsR, MazF, RelE, ChpB, YoeB, and YafQ prevent translation by cleaving RNAs (Zhang et al., 2003; Christensen et al., 2004; Gerdes et al., 2005; Brown et al., 2009; Prysak et al., 2009).

The role of TA systems are becoming less enigmatic and they have been implicated in several functions among which include, gene regulation, control of growth, persister cell formation, programmed cell arrest, programmed cell death, and anti-phage measures (Gerdes et al., 2005; Magnuson, 2007; Van Melderen and Saavedra De Bast, 2009). The role of TA systems in biofilm formation has been demonstrated as well as it is currently recognized that these systems can direct cells toward the formation of biofilm and persister cells (Kim and Wood, 2010). In mutant strains of *E. coli* lacking TA pairs MazF/MazE, RelE/RelB, YoeB/YefM, YafQ/DinJ and ChpB, biofilm formation has been demonstrated to be affected (Kim et al., 2009, 2010; Kolodkin-Gal et al., 2009). TA systems in cryptic prophages have been found to influence biofilm formation, e.g., deletion of toxin YpjF from the TA pair YpjF-YfjZ of the cryptic prophage CP4-57 of *E. coli* K-12 increased biofilm formation (Brown and Shaw, 2003; Wang et al., 2009, 2010).

The MqsR/MqsA pair of *E. coli*, was the first TA system found to be associated with biofilm formation when *mqsR* was identified among genes that were differentially regulated in biofilm cells (Ren et al., 2004). This system is conserved in 40 eubacteria and in many genera such as *Y. pseudotuberculosis*, *Y. pestis*, *Bordetella bronchiseptica*, and *P. fluorescens* (Kim et al., 2010). The toxin MqsR is an RNase belonging to the RelE family that cleaves mRNA at GCU and, to a lesser extent, GCA sequences. The antitoxin MqsA, which is located immediately downstream of *mqsR* in the same operon, is a DNA/binding protein that functions to neutralize MqsR toxicity (Brown et al., 2009). It has been shown that a deletion of *mqsRA* reduces biofilm formation in *E. coli* and this effect is due to MqsR favoring biofilm formation by affecting cellular motility through autoinducer-2 signaling (Gonzalez Barrios et al., 2006; Kasari et al., 2010). Furthermore, it also plays a role in *E. coli* biofilm and persistence by positively regulating the expression of toxin CspD and QseBC, the two component motility regulatory system (Gonzalez Barrios et al., 2006; Kim et al., 2010). On another level, MqsA is able to bind to its regulatory palindromic sequence found upstream of the CsgD promoter region, preventing CsgD transcription which in turn decreases curli biosynthesis and *E. coli* biofilm formation (Soo and Wood, 2013). Interestingly, this system is subject to an auto-post-transcriptional control, as free MqsR has the ability to degrade its own mRNA, and that of *mqsA*, thus alleviating the persister state and biofilm formation once the environmental stress is removed (Brown et al., 2013).

MqsRA can favor biofilm formation by indirectly controlling the level of c-di-GMP. This effect is the result of a mix of transcriptional and post-transcriptional mechanisms which start with the detection of stress conditions. In such conditions, the protease Lon degrades MqsA which acts as a negative regulator of *rpoS* by binding directly to its promoter (Wang et al., 2011). Once MqsA is degraded, the sigma factor encoded by *rpoS*, which controls up to 500 genes in *E. coli* (Hengge, 2008), is induced. This systematically leads to increases in expression of genes involved in c-di-GMP synthesis and repression of *flhDC* genes, leading to inhibition of motility, and induction of *csgD*, favoring both curli and cellulose production (Pesavento et al., 2008). Together all of these processes enhance the formation of biofilm (Wang et al., 2011).

Therefore, given that toxins are mRNA interferases that degrade mRNA with substrate specificity, they can be viewed as acting similarly to the global regulators like Hfq and CsrA or RNases (described below) that regulate gene expression at a post-transcriptional level by modulating mRNA decay.

### RNAses

RNases are enzymes that cleave RNAs, resulting in remarkably diverse biological consequences. Their elucidated role in biofilm production is mainly that of causing decay of ncRNAs that are involved in regulating this process. The primary endonuclease involved in mRNA decay is RNase E. The RNA-binding protein CsrA activity is indirectly regulated by RNase E. The expression of the two ncRNAs, CsrB and CsrC, that regulate CsrA activity in *E. coli* are regulated by the CsrD protein, which controls their degradation. This CsrD-mediated RNA decay requires RNase E, as demonstrated by the increased half-life of both CsrB and CsrC in *rnaseE* mutants. Despite CsrD containing a GGDEF/EAL motif, its function does not involve c-di-GMP synthesis or turnover (Suzuki et al., 2006). These results indicate that RNase E plays an important role in post-transcriptionally mediating biofilm formation, by degrading ncRNAs CsrB and CsrC (Suzuki et al., 2006).

Another RNase that acts similarly to degrade ncRNAs is RNase G which is implicated in mRNA maturation and turnover processing in *E. coli*. In *P. aeruginosa*, RNase G is involved in biofilm production by controlling the levels of the ncRNA RsmZ. In these bacteria, transition to later stages of biofilm formation is regulated by three two component regulatory systems, BfiSR, BfmSRR, and MifSR (Petrova and Sauer, 2009). BfiSR is required for transition to the irreversible attachment stage and it has been shown that it regulates biofilm development via the degradation of the ncRNA RsmZ through RNase G (CafA) (Petrova and Sauer, 2009, 2010). BfiR binds directly upstream *cafA*, encoding the RNase G, to activate its expression and once expressed, RNase G targets RsmZ under biofilm growth conditions. In *P. aeruginosa*, reduced RsmZ levels are essential to form biofilm. Thus, when *cafA* is inactivated, an increase in RsmZ levels occurs and a subsequent reduction in biofilm formation (Petrova and Sauer, 2010).

Rnase Y was found to regulate cleavage of the mRNAs of genes involved in biofilm formation in *B. subtilis*. Here, increasing quantities of the mRNA of the biofilm repressor gene *sinR* were observed with depletion of Rnase Y, resulting in less biofilm formation. Reciprocally, there appeared to be a correlation between increasing amounts of mRNAs of genes required for biofilm and concomitant increased biofilm production, with over-expression of the *rny* gene, encoding RnaseY (Lehnik-Habrink et al., 2011).

### 3'-untranslated region (3'-UTR)

Although it is well known that 3'-UTRs of mRNA molecules in eukaryotes impact mRNA stability and translational efficiency, such experimental knowledge has been lacking for bacteria. However, Ruiz de los Mozos et al recently found that one third of the mapped mRNAs of *S. aureus* contains long 3'-UTRs (>100 nucleotides) (Ruiz de Los Mozos et al., 2013). Their specific investigation of the regulatory role of the long 3'-UTR of the *icaR* mRNA which codes for the repressor of the main exopolysaccharide compound of the *S. aureus* biofilm matrix, indicated that this 3'-UTR can base-pair with the SD sequence of the *icaR* mRNA and interfere with the translation initiation complex. This results in formation of a double stranded DNA substrate for RNaseIII and subsequent inhibition of biofilm development in *S. aureus*. This is a first report of such a mechanism involved in biofilm formation and further investigations into the presence of long 3'-UTRs in mRNAs of genes required for biofilm in other biofilm producing bacteria could reveal that this is a conserved mechanism of post-transcriptional regulation.

## Conclusions

Biofilm formation and development is a fascinatingly intricate process involving finely altered gene expression, requiring complex and well-coordinated regulation to accomplish the process with high efficiency both spatially and temporally. In this review we have exemplified several of the well characterized powerful contributions that post-transcriptional regulation makes to rapidly adjust and fine tune gene expression to the developmental needs of the cell during biofilm formation. These mechanisms confirm that bacterial signal integration and gene regulation at the mRNA level might be equally sophisticated as its transcription-factor based counterpart acting at the DNA level, with 5' UTRs of mRNAs playing an analogous role to that of complex promoters. It is however clear that with the growing body of discoveries about the complexities of post-transcriptional regulation we should expect that many new pathways and molecules play critical roles in biofilm formation. This serves as grounds for encouragement for the continued surge into biofilm regulation research which will definitely shed more light on the complex intricacies of this biological process.

## Author contributions

Luary C. Martínez reviewed the literature and contributed to writing and revising this manuscript. Viveka Vadyvaloo contributed to writing and revising this manuscript.

### Conflict of interest statement

The authors declare that the research was conducted in the absence of any commercial or financial relationships that could be construed as a potential conflict of interest.
